# Supplementary material for: Ultrasonic Treatment of Corn Starch to Improve the Freeze-Thaw Resistance of Frozen Model Dough and Its Application in Steamed Buns
Source: Foods. 2023 May 12;12(10):1962. doi: 10.3390/foods12101962 (PMC10217374; doi:10.3390/foods12101962)
Supplement: Supplementary file 1 [file foods-12-01962-s001.zip › foods-2381565-supplementary.pdf]

foods

MDPI

Supplementary Materials:

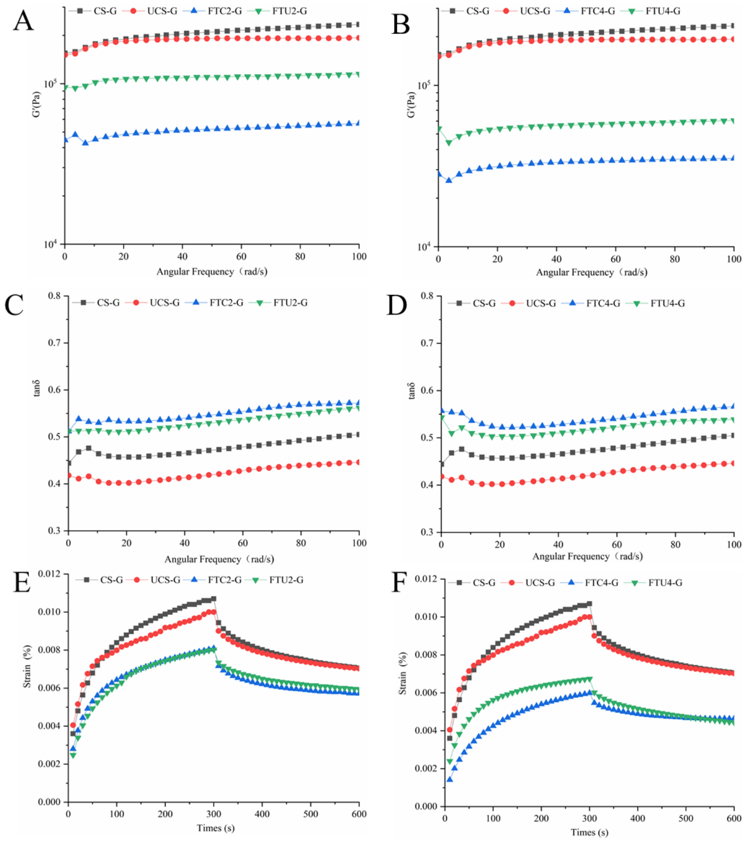

Figure S1. The elastic modulus, loss angle tangent and creep recovery curves of ultrasonically modified corn starch model dough at 2,4 freeze-thaw cycles. A-B is the elastic modulus, C-D is the loss angle tangent and E-F is the creep recovery curve.

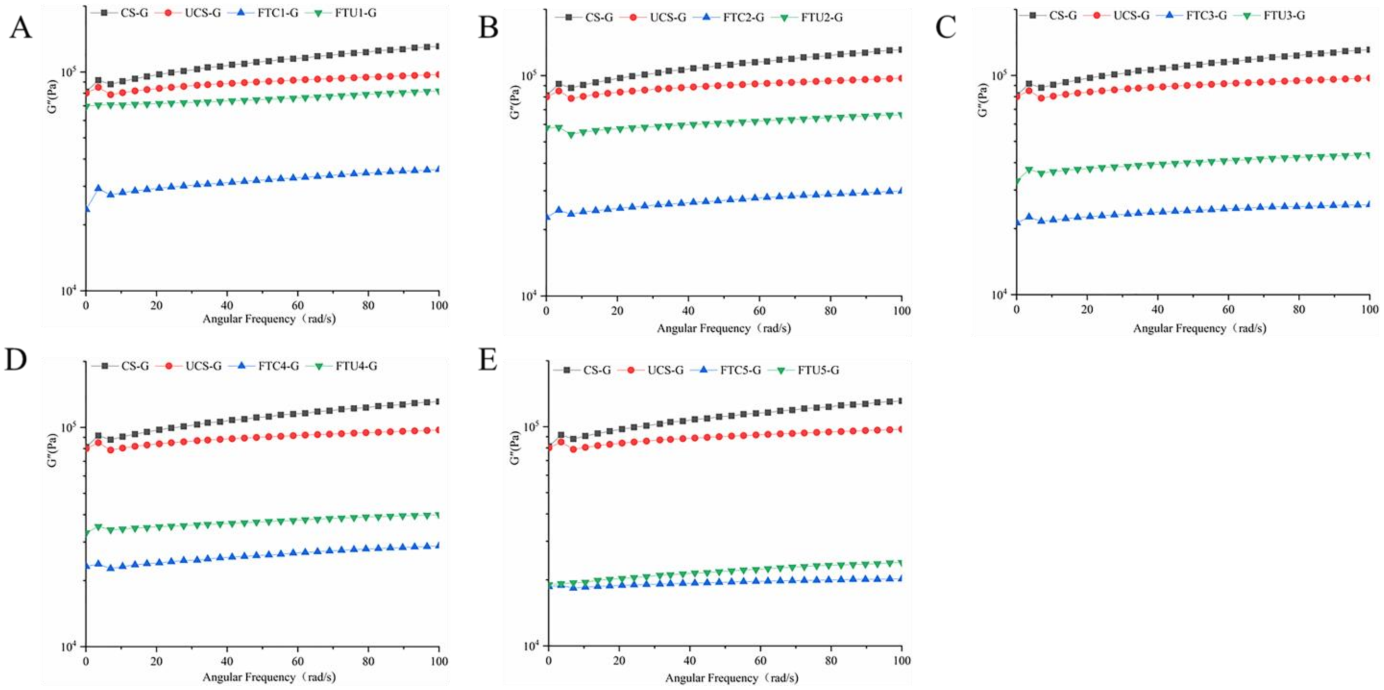

Figure S2. Viscosity modulus of ultrasonically modified corn starch model doughs under different number of freeze-thaw cycles

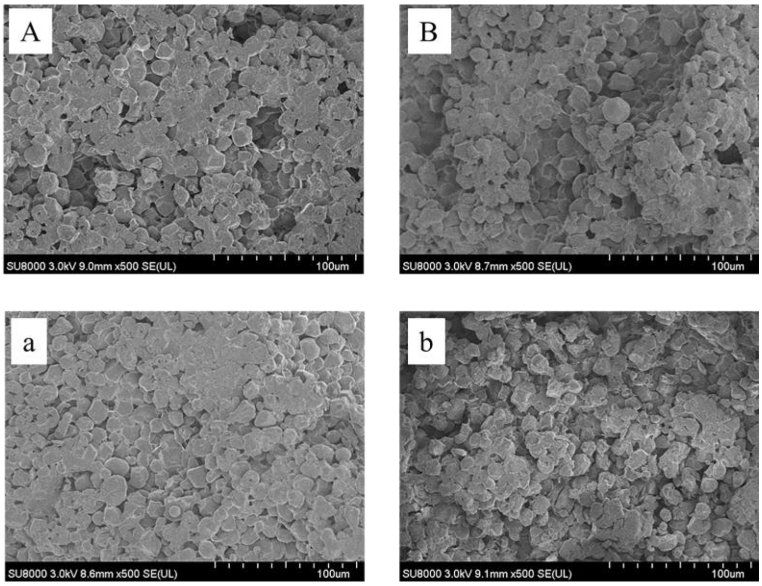

Figure S3. Scanning electron micrographs of ultrasonically modified corn starch model dough at 2,4 freeze-thaw cycles.
